# Tumor Treating Fields (TTFields) therapy vs physicians’ choice standard-of-care treatment in patients with recurrent glioblastoma: a post-approval registry study (EF-19)

**DOI:** 10.1007/s12672-022-00555-5

**Published:** 2022-10-14

**Authors:** Jay-Jiguang Zhu, Samuel A. Goldlust, Lawrence R. Kleinberg, Jérôme Honnorat, Nancy Ann Oberheim Bush, Zvi Ram

**Affiliations:** 1 University of Texas Health Science Center in Houston (UTHealth)/Memorial Hermann Hospital at Texas Medical Center, 6400 Fannin St., Suite 2800, Houston, TX 77030 USA; 2 John Theurer Cancer Center, Hackensack, NJ USA; 3 Department of Radiation Oncology and Molecular Radiation Sciences, Johns Hopkins University School of Medicine, Baltimore, MD USA; 4 Department of Neuro-Oncology, Hôpital Neurologique, Hospices Civils de Lyon, SynatAc Team, MELIS Institute, INSERM U1314/CNRS UMR5284, Université Claude Bernard Lyon 1, Université de Lyon, Lyon, France; 5 Department of Neuro-Oncology, East Group Hospital, Hospices Civils de Lyon, Lyon Cedex, France; 6 Department of Neurological Surgery and Neurology, University of California, San Francisco, CA USA; 7 Tel Aviv Medical Center, Tel Aviv University School of Medicine, Tel Aviv, Israel

## Abstract

**Purpose:**

Tumor Treating Fields (TTFields) therapy, a noninvasive, anti-mitotic treatment modality, is approved for recurrent glioblastoma (rGBM) and newly diagnosed GBM based on phase III, EF-11 (NCT00379470) and EF-14 (NCT00916409) studies, respectively. The EF-19 study aimed to evaluate efficacy and safety of TTFields monotherapy (200 kHz) vs physicians’ choice standard of care (PC-SOC; EF-11 historical control group) in rGBM.

**Methods:**

A prospective, post-marketing registry study of adults with supratentorial rGBM treated with TTFields therapy was conducted. Primary endpoint was overall survival (OS; intent-to-treat [ITT] population) and secondary endpoint was OS per-protocol (PP). Subgroup and toxicity analyses were conducted.

**Results:**

Median OS (ITT population) was comparable with TTFields monotherapy vs PC-SOC (7.4 vs 6.4 months, log-rank test *P* = 0.053; Cox test hazard ratio [HR] [95% CI], 0.66 [0.47–0.92], *P* = 0.016). The upper-bound HR (95% CI) was lower than pre-defined noninferiority (1.375 threshold). In the PP population, median OS was significantly longer for TTFields monotherapy vs PC-SOC (8.1 vs 6.4 months; log-rank test *P* = 0.017; Cox test HR [95% CI], 0.60 [0.42–0.85], *P* = 0.004). TTFields therapy showed increased benefit with extended use (≥ 18 h/day [averaged over 28 days]). TTFields therapy-related adverse events (AEs) by body system were lower vs PC-SOC: mainly mild-to-moderate skin AEs.

**Conclusion:**

In the real-world setting, TTFields monotherapy showed comparable (ITT population) and superior (PP population) OS vs PC-SOC in rGBM. In line with previous results, TTFields therapy showed a favorable safety profile vs chemotherapy, without new safety signals/systemic effects.

*Trial registration*: NCT01756729, registered December 20, 2012.

**Graphical Abstract:**

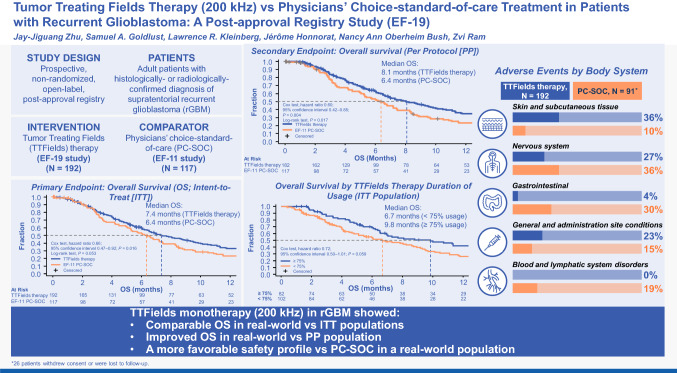

**Supplementary Information:**

The online version contains supplementary material available at 10.1007/s12672-022-00555-5.

## Introduction

Glioblastoma (GBM; grade-IV isocitrate dehydrogenase-1 [IDH1] wildtype glioma [1, 2]) is the most common locally invasive and aggressively infiltrative primary malignant brain tumor [3–6], making total resection implausible.

Historically, standard adjuvant therapy in newly diagnosed GBM (ndGBM) has been based on the Stupp protocol, comprising maximal safe resection/biopsy, followed by radiation therapy [RT] with concomitant and 6–12 months maintenance temozolomide (TMZ) [7].

In contrast, optimal management of patients with recurrent GBM (rGBM) has traditionally been less well defined. Recommended treatment options include craniotomy with/without carmustine implant, laser interstitial thermal therapy (LITT) [8], stereotactic radiosurgery [9], or salvage RT followed by systemic therapy [10], (i.e., TMZ [11], nitrosoureas [e.g., carmustine or lomustine] [12, 13], and bevacizumab [14]). However, once tumors progress after first-line therapy, treatment options are limited and results are often unsatisfactory, making management of rGBM a considerable challenge [15].

Tumor Treating Fields therapy (TTFields, Optune^®^; Novocure.^®^ GmbH; device manufacturer) is a first-in-class, noninvasive, loco-regional cancer treatment approved for use in ndGBM, rGBM and malignant pleural mesothelioma [16, 17]. TTFields are electric fields generated by a portable medical device that is designed to be integrated into daily life, while maintaining patients’ quality of life, and delivered via scalp-placed arrays [2, 18]. TTFields work by disrupting normal localization and function of polar components within cells, and specifically target cancer cells due to characteristics that distinguish them from healthy cells and tissue [19–24]

Since its approval in the European Union (EU), US, Japan, and China [16, 25–28], TTFields therapy concomitant with maintenance TMZ has been incorporated into the GBM treatment paradigm, expanding treatment options for this patient population with high unmet needs. Additionally, the National Comprehensive Cancer Network guidelines recommend TTFields therapy (category 1 recommendation) for adult patients with ndGBM [29, 30].

Results from the EF-14 (NCT00916409) study formed the basis for approval for use in ndGBM. EF-14 demonstrated significantly improved progression-free survival (PFS) for TTFields therapy concomitant with TMZ (6.7 months) vs TMZ alone (4.0 months) and a significantly improved overall survival (OS) for TTFields therapy concomitant with TMZ (20.9 months) vs TMZ alone (16.0 months) [31]. Additionally, 5-year OS rates were more than double that of TMZ alone (5% vs 13%; *P* = 0.04) [31].

Additionally, the EF-11 study, in which TTFields therapy was compared with best standard of care (SOC) in patients with rGBM, also showed improvements in outcomes and formed the basis for approval in rGBM [32]. TTFields therapy has also shown clinical efficacy in a range of other solid tumors, including non-small cell lung cancer, liver, ovarian, and pancreatic cancer, when used concomitantly with systemic therapies and alongside radiation [33–38]. Clinical and real-world data demonstrate that TTFields therapy has a favorable safety profile, characterized by a low rate of systemic adverse events (AEs) compared with chemotherapeutic regimens. AEs related to TTFields therapy are predominantly dermatologic in nature and can be managed with appropriate prophylaxis and intervention [31, 32, 39–41].

Here we report the results of a prospective, open-label, multi-site, post-marketing registry study of patients with rGBM treated with TTFields monotherapy (200 kHz), evaluating efficacy and safety vs the physicians’ choice SOC (PC-SOC) EF-11 study cohort arm (historical control group).

## Methods

### Study design

The EF-19 (NCT01756729) study was a prospective, open-label, multi-site, post-marketing registry study designed to compare the efficacy and safety of TTFields monotherapy in patients with rGBM to PC-SOC (historical control group) from the EF-11 study. The recruitment period was approximately 24 months and follow-up period was 12 months from last patient recruited. Patient follow-up was conducted according to standard practices at each participating center; evaluation of AEs, vital signs, and Karnofsky Performance Status (KPS) (where available) were performed at each follow-up appointment. Patients were also seen by a Novocure-trained Device Support Specialist (DSS) once per month during follow-up.

### Study population

The treatment population was comprised of patients already enrolled in the PRiDe registry (which includes all patients in the US treated with TTFields therapy, commercially), therefore, patients were recruited to this study from either the outpatient clinic or inpatient hospital setting at any certified TTFields therapy center in the US. All patients provided written consent for use of their data in the registry, including protected health information.

Full inclusion and exclusion criteria are listed in the supplementary material. Adult patients (≥ 22 years of age) with a histologically or radiologically confirmed diagnosis of supratentorial rGBM, per Macdonald’s criteria [25] with KPS score of  ≥ 70 enrolled in the PRiDe registry were eligible. The control arm comprised patients from the PC-SOC cohort of the EF-11 study [32].

### Study treatment

Continuous TTFields (200 kHz) were generated using the NovoTTF-100A system and delivered for ≥ 18 h/day, for a minimum of 4 weeks, through scalp-placed arrays which remained in place during treatment (arrays could be removed during treatment breaks and during routine array changes). Patients carried/wore the field generator and battery in a bag connected to the arrays whilst receiving treatment, as these components are essential for the generation of TTFields. Before TTFields therapy was initiated, patients/caregivers were provided with education and training on TTFields therapy usage by certified physician/nurse staff. To educate patients and caregivers on operating the device independently, access to a designated DSS (service by device manufacturer, Novocure^®^) and hotline assistance (24 h/day) were provided to patients for additional technical support. All treatment was delivered on an outpatient basis. Arrays were supplied in individual sterile packages for hygienic application. Array changes (~ twice per week) were conducted by patient, caregiver(s), and/or a DSS. TTFields therapy continued until clinical disease progression (new central nervous system symptoms/worsening of symptoms and significant radiological tumor growth), development of significant/serious AEs or patient withdrawal.

### Assessments and outcomes

The primary efficacy endpoint was OS (months) from time of treatment initiation in the intent-to-treat (ITT) population, evaluating noninferiority of TTFields monotherapy vs PC-SOC. The key additional endpoint was OS (months) in the per-protocol (PP) population, evaluating noninferiority vs PC-SOC. Furthermore, the additional endpoint of median time to treatment failure (months), defined as time from treatment start to treatment discontinuation, in patients treated with TTFields therapy vs PC-SOC was assessed in the ITT population, serving as a surrogate measure for progressive disease (PD). Other additional endpoints in the ITT population included incidence of AEs and OS according to O[6]-methylguanine-DNA methyltransferase (*MGMT*) promoter methylation and *IDH1* gene mutation statuses in the ITT population. PD was not evaluated as an EF-19 endpoint and served as a guide to assess any needs for treatment cessation of TTFields therapy.

AE information was collated during the study period from unsolicited patient/caregiver/investigator reports, during interactions with DSS/prescribers, and from nCompass™ support emails, and was processed according to routine post-market surveillance activities from the manufacturer. AEs were classified using Medical Dictionary for Regulatory Activities dictionary version 22.1 (MedDRA v22.1). Due to the nature of this registry, AE severity could only be classified as non-serious or serious.

### Statistical analyses

All analyses were performed using Statistical Analysis System (SAS), version 9.4 (SAS Institute Inc, Cary, NC, USA).

A sample size of 192 patients in the TTFields arm (including estimated 10% loss to follow-up rate) was determined, based on a noninferiority *log-rank test* with a 2-sided alpha level of 0.05 and a power of 80%, comparing time to event (i.e., death) in patients treated with TTFields therapy and PC-SOC. The sample size analysis was performed using NCSS-PASS 11 16.0 (NCSS, Kaysville, UT, USA). The null hypothesis was that the hazard-of-death would be higher for TTFields monotherapy (hazard ratio [HR] > 1.375); and alternative hypothesis HR would be ≤ 1.375. An HR of 0.1083 with PC-SOC was estimated from the rate-of-death per month in the PC-SOC arm. For OS analyses, patients without a known date of death were censored at last known date documented alive. Analyses were conducted under the assumptions that the active treatment and control arms had equivalent background characteristics, and that treatments were applied contemporaneously, even when they were not.

Additional analyses of annual OS rates were compared between groups, using a 1-sided Z distribution of the Kaplan–Meier estimates of OS rates at the defined timepoint. Cox proportional hazards model was used to analyze OS, controlling for treatment group, age, sex, KPS and type of rGBM resection. Threshold for significant interactions was specified at an alpha of 0.05.

The ITT population included all enrolled patients from the TTFields therapy and historical control arms. The PP population included patients who received ≥ 28 days of TTFields monotherapy for ≥ 18 h/day (on average in the first month of treatment) and all PC-SOC patients who received systemic therapy.

## Results

### Baseline characteristics and patient disposition

Overall, 1082 patients with rGBM who received TTFields monotherapy in the US clinical-practice setting from February 10, 2016 to December 28, 2017 were screened for eligibility. Of these, 192 patients met preset EF-19 registry study inclusion criteria for enrolment at TTFields therapy-certified US sites. Patients were enrolled and cared for by 151 unique certified US-site physicians. The control arm included 117 patients from the PC-SOC cohort of the EF‐11 study.

Key baseline characteristics were comparable between groups, including age, sex, KPS score, and extent of surgical resection at diagnosis (Table 1). Of patients who had available tissue samples (84/192 [44%] patients in the TTFields therapy arm) for genomic evaluation, 42% had *MGMT* promoter methylation. Data on *MGMT* and *IDH1* status were not collected in the EF-11 study.

**Table 1:** Baseline characteristics and patient disposition in TTFields therapy EF-19 registry and EF-11 PC-SOC arms

Baseline characteristics and patient disposition	TTFields therapy EF-19 Registry (N = 192)	PC-SOC EF-11 (N = 117)
Age, median (min–max) years of age	57.0 (23–80)	54.0 (29–74)
Sex, n (%)		
Male	125 (65)	73 (62)
Female	67 (35)	44 (38)
KPS score, n (%)	129 (67)	114 (97)
Median (min–max)	80.0 (70–100)	80.0 (50–100)
Race, n (%)	164 (85)	117 (100)
White	141 (73)	106 (91)
African American	9 (5)	5 (4)
Asian	6 (3)	3 (3)
Hispanic	8 (4)	2 (2)
Other	0	1 (1)
Extent of initial resection, n (%)	189 (98)	NA
Biopsy	15 (8)	NA
Partial resection	44 (23)	NA
Gross total resection	130 (69)	NA
Extent of resection at time of recurrence, n (%)	183 (95)	117 (100)
None	106 (55)	88 (75)
Biopsy	4 (2)	0 (0)
Partial resection	10 (5)	3 (3)
Gross total resection	63 (33)	26 (22)
*MGMT* promoter methylation status, n (%)	84 (44)	NA
*MGMT* promoter methylation status	35 (42)	NA
No *MGMT* promoter methylation status	49 (58)	NA
*IDH1* gene status, n (%)	93 (48)	NA
Mutated *IDH1* gene status	16 (17)	NA
Wild-type *IDH1* gene status	77 (83)	NA
Number of prior-chemotherapy lines, n (%)	190 (99)	NA
Median (min–max)	2 (0–7)	NA
0	4 (2)	NA
1	96 (51)	NA
2	60 (32)	NA
3	20 (11)	NA
> 3	10 (5)	NA
Steroid usage, n (%)	85 (44)	44 (38)
Anticonvulsant usage^a^, n (%)	142 (74)	50 (43)
ITT population, N (%)	192 (100)	117 (100)
Remained on treatment at study closure, n (%)	8 (4)	NA
Treatment discontinuations, n (%)	184 (96)	NA
Due to death^b^	134 (70)	NA
Other reasons^c^	50 (26)	NA
PP population^d^, N (%)	182 (95)	117 (100)

Percent values have been rounded to nearest integer

*“NA”* represents variables that were not collected in the EF-11 clinical study, therefore no comparable data are available [32]

*ITT* intent-to-treat; *IDH1* isocitrate dehydrogenase 1; *KPS* Karnofsky Performance Status; *MGMT* O6-Methylguanine-DNA-methyltransferase; *MRI* magnetic resonance imaging; *NA* not available; *PC-SOC* physicians’ choice standard of care; *PD* progressive disease; *PP* per-protocol; *TTFields* Tumor Treating Fields

^a^Anticonvulsant use, during the EF-11 study, was recorded by study teams using clinical case report forms. In the post-approval EF-19 registry study, recording was done using patient charts. Any evidence of anticonvulsant use in the charts of each registry patient was recorded, as it was not possible to determine start and stop dates. Therefore, the number of patients noted in the TTFields therapy EF-19 registry arm does not reflect patients who used anticonvulsants at baseline, but rather the number of patients with any past or current use of anticonvulsants

^b^Death-related reasons leading to treatment discontinuations in the ITT population of TTFields therapy EF-19 registry, n (%), were due to: underlying disease, 88 (46); pneumonia respiratory failure, 1 (1); cardiopulmonary arrest, 1 (1); and unknown causes, 44 (23)

^c^Other reasons leading to treatment discontinuations in the ITT population of EF-19 TTFields therapy registry arm, n (%), were due to: PD, 14 (7); other medical reasons, 8 (4); patient decision, 14 (7); physician’s decision, 7 (4); MRI scan did not show tumor, 1 (1); and unknown reasons, 6 (3)

^d^PP population defined as patients who received ≥ 28 days of TTFields monotherapy (200 kHz) for ≥ 18 h/day on average in the first month of treatment and all PC-SOC patients

The disposition of patients treated with TTFields therapy in the EF-19 registry study is summarized in Table 1. All patients treated with PC-SOC (N = 117) received systemic therapy, either during or after the EF-11 study, and were included in the PC-SOC PP population [32]. Of these patients, 26 (22%) withdrew consent and were lost to safety follow-up, for which only survival data was collected. Safety data are presented for the remaining 91 (78%) patients in the PC-SOC control arm.

### Efficacy outcomes

#### Primary efficacy outcome

**Fig. 1:**
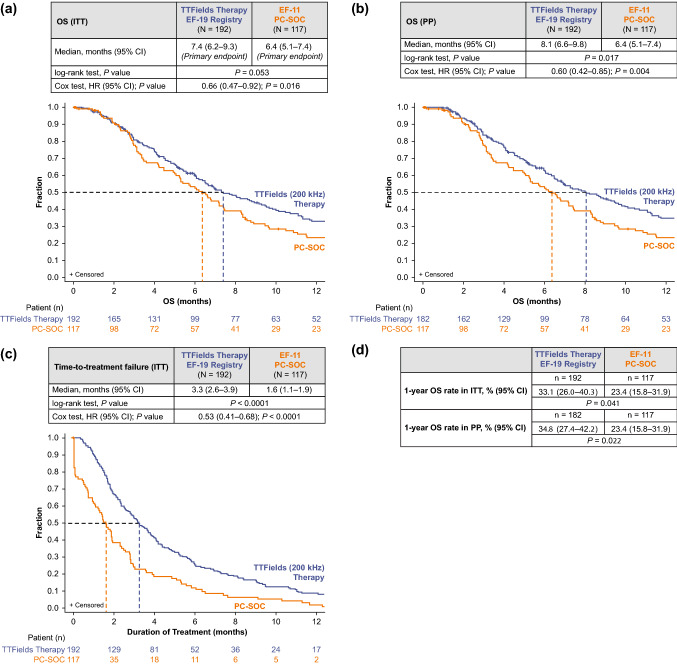
Kaplan–Meier curve comparing TTFields (200 kHz) therapy from EF-19 registry and PC-SOC patients with rGBM for **a** OS in ITT populations (primary endpoint), **b** OS in PP populations, **c** time to treatment failure in ITT populations, and **d** 1-year OS rates in ITT and PP populations. CI, confidence interval; HR, hazard ratio; ITT, intent-to-treat; OS, overall survival; PC-SOC, physicians’ choice standard of care; PP, per protocol; rGBM, recurrent glioblastoma; TTFields, Tumor Treating Fields

In the ITT population, OS (95% CI) was 7.4 months (range 6.2–9.3) with TTFields therapy vs 6.4 months (range 5.1–7.4) with PC-SOC (*P* = 0.053, *log-rank test*; Fig. 1A). The Cox proportional HR was 0.66 (95% CI 0.47–0.92; *P* = 0.016, *Cox test*; Fig. 1A). The primary endpoint was met (HR 95% CI upper limit was ≤ 1.375, pre-defined threshold). The statistically significant *Cox test* for HR of 0.66 (*P* = 0.016) indicated that TTFields therapy reduced risk of death by 34% vs PC-SOC.

#### Additional efficacy outcomes

In the PP population, OS (95% CI) was 8.1 months (range 6.6–9.8) with TTFields therapy vs 6.4 months (range 5.1–7.4) with PC-SOC (*P* = 0.017, *log-rank test*; Fig. 1B). The Cox proportional HR for OS (95% CI) was 0.60 (0.42–0.85), showing statistical significance (*P* = 0.004, *Cox test*; Fig. 1B).

Median (95% CI) time to treatment failure in the ITT population was 3.3 months (range 2.6–3.9) with TTFields therapy and 1.6 months (range 1.1–1.9) with PC-SOC (*P* < 0.0001, *log-rank test*; Fig. 1C). The Cox proportional HR (95% CI) for time to treatment failure was 0.53 (0.41–0.68) for TTFields therapy vs PC-SOC (*Cox test P* < 0.0001; Fig. 1C).

Moreover, OS rate (95% CI) at 1 year in the ITT population was 33% (26–40) with TTFields therapy and 23% (16–32) with PC-SOC (*P* = 0.041). The OS rate (95% CI) in the PP population was 35% (27–42) with TTFields therapy and 23% (16–32) with PC-SOC (*P* = 0.022, *log-rank test*; Fig. 1D)**.** Improved OS rate at 1 year was ~ 1.5 times greater in both ITT and PP populations with TTFields therapy vs PC-SOC.

### Subgroup analyses

**Fig. 2:**
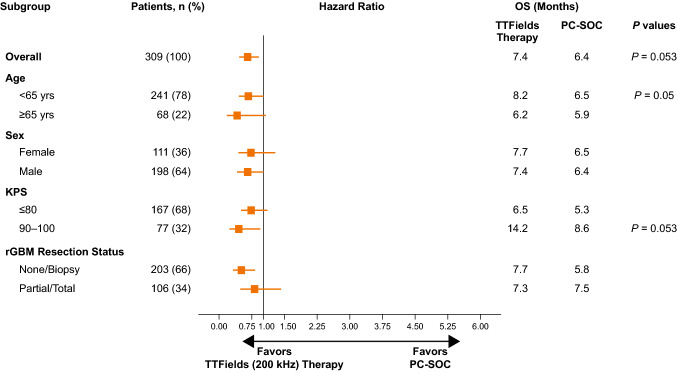
Cox hazard ratios of OS by prognostic factor subgroups in EF-19 registry of TTFields therapy (N = 192) and PC-SOC (N = 117) in ITT population for patients with rGBM. Symbols represent Cox HRs in each subgroup of patients treated with TTFields therapy in EF-19 registry compared to PC-SOC arm (EF-11 clinical study control group), while adjusting for other prognostic factors. Whiskers represent the 95% CIs of the HR. CI, confidence interval; HR, hazard ratio; KPS, Karnofsky Performance Status score; OS, median overall survival; PC-SOC, physicians’ choice standard of care; rGBM, recurrent glioblastoma; TTFields, Tumor Treating Fields

In additional analyses, TTFields monotherapy was associated with a statistically significant increase in OS overall, and within certain patient subgroups in the ITT population (Fig. 2; Cox proportional hazards, *P* < 0.05 for the treatment effect within each subgroup). Clinical benefit of TTFields therapy on OS was observed regardless of KPS, age, sex, and rGBM resection status. Similar findings were obtained when patients were stratified by rGBM resection status—none/biopsy/partial resection vs those with gross total resection (data not shown).

**Fig. 3:**
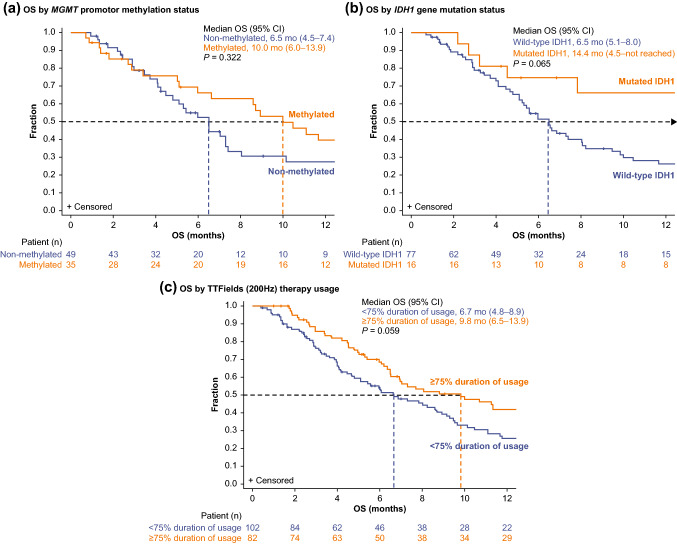
Comparison of EF-19 registry of patients with rGBM treated with TTFields (200 kHz) therapy in the ITT population by subgroup analyses: **a** *MGMT* promotor methylation status (n = 84), **b** *IDH1* gene mutation status (n = 93), and **c** duration of TTFields treatment usage (n = 184). CI*,* confidence interval; *IDH1,* isocitrate dehydrogenase 1; *MGMT,* O6-Methylguanine-DNA-methyltransferase; OS, overall survival; rGBM, recurrent glioblastoma; TTFields, Tumor Treating Fields

In the TTFields therapy arm, patients whose tumors were *MGMT* promoter methylation status positive had a numerically extended OS (95% CI) of 10 months (6.0–13.9) vs 6.5 months (4.5–7.4) in patients with tumor-negative status; HR (95% CI) was 0.77 (0.46–1.30), (*P* = 0.322, *log-rank test*; Fig. 3A).

Patients with *IDH1* gene mutation in the ITT population demonstrated a trend toward longer OS (95% CI) of 14.4 months (4.5–not reached) vs 6.5 months (5.1–8.0) in patients with wild-type *IDH1* gene status; HR (95% CI) of 0.52 (0.25–1.05; *P* = 0.065 Fig. 3B). These tumor biomarker subgroup assessments (*IDH1* and *MGMT*) were not performed in the EF-11 study (non-comparable).

In the TTFields arm, patients who were treated for ≥ 18 h/day (≥ 75% daily usage; monthly average in the first 6 months of treatment) in the ITT population had a longer OS (95% CI) of 9.8 months (6.5–13.9) vs 6.7 months (4.8–8.9) for < 18 h/day usage (< 75% daily usage, monthly average in the first 6 months of treatment); HR (95% CI) of 0.72 (0.50–1.01), *P* = 0.059, *log-rank test* (Fig. 3C). Moreover, treatment with TTFields therapy for ≥ 18 h/day usage vs PC-SOC was positively correlated with higher survival rates, respectively, at 6 (69% vs 52%; *P* = 0.011), 12 (42% vs 23%; *P* = 0.005), 15 (34% vs 22%; *P* = 0.049), and 18 (27% vs 18%; *P* = 0.091) months.

### Adverse events

Safety data for all 192 registry patients with a database cut-off date of December 31, 2018 were compared descriptively to the 91 PC-SOC patients. The number of patients with any reported AE ≥ 1 was lower with TTFields therapy vs PC-SOC (67% vs 95%, respectively) in the safety population (N = 283) (Table 2). Overall, PC-SOC treatment demonstrated higher AE incidence rates (≥ 5% incidence in any arm) compared to TTFields monotherapy across 12 of 15 classifications by MedDRA body system organ class (Table 2). For the 12 AE classes with greater AE incidence with PC-SOC, the most commonly reported AE by body system class in the PC-SOC arm was nervous system disorders (36%), followed by gastrointestinal disorders (30%) and blood and lymphatic system disorders (19%). In contrast, AE incidence rates were greater in the TTFields monotherapy vs PC-SOC group for 3 AE classifications, which were: skin and subcutaneous tissue disorders (36%); general disorders and administration-site conditions (23%); and injury, poisoning, and procedural complications category (11%). There were no skin AEs in the EF-19 registry that resulted in TTFields therapy discontinuation. Reported skin AEs were deemed as non-serious, consistent with results from the previously reported EF-11 study.

**Table 2:** Number of patients with adverse events by body system (≥ 5% incidence in any arm)

MedDRA version 22.1 Body system, n (%)	TTFields therapy EF-19 Registry (N = 192)	PC-SOC EF-11 (N = 91)
Number of patients with ≥ 1 any AE	128 (67)	86 (95)
Blood and lymphatic system disorders	0 (0)	17 (19)
Cardiac disorders	1 (1)	6 (7)
Eye disorders	0 (0)	5 (5)
Gastrointestinal disorders	8 (4)	27 (30)
General disorders and administration-site conditions	45 (23)	14 (15)
Infection and infestations	6 (3)	11 (12)
Injury, poisoning, and procedural complications	22 (11)	1 (1)
Investigations	0 (0)	5 (5)
Metabolism and nutrition disorders	1 (1)	12 (13)
Musculoskeletal and connective tissue disorders	8 (4)	8 (9)
Nervous system disorders	51 (27)	33 (36)
Psychiatric disorders	7 (4)	7 (8)
Respiratory, thoracic and mediastinal disorders	2 (1)	10 (11)
Skin and subcutaneous tissue disorders	70 (36)	9 (10)
Vascular disorders	2 (1)	6 (7)

Percent values have been rounded to nearest integer

*AEs* adverse events; *MedDRA* Medical Dictionary for Regulatory Activities; *PC-SOC* physicians’ choice standard of care; *TTFields* Tumor Treating Fields

## Discussion

This was a post-marketing registry study conducted in the real-world setting in patients with rGBM. This study was conducted to provide further evidence to support the efficacy and tolerability of TTFields therapy in rGBM following the phase III EF-11 clinical study, which underpinned approval in the EU, US, Japan, and China [16, 25–28]. Data from this study confirmed that TTFields monotherapy (200 kHz) was noninferior to PC-SOC in OS compared with EF-11 study data [32]. OS in TTFields monotherapy patients was comparable to PC-SOC in the ITT population (primary endpoint), with a more favorable safety profile. After a minimum follow-up of 12 months, OS was 7.4 vs 6.4 months for the TTFields therapy vs PC-SOC arms, respectively (HR [95% CI], 0.66 [0.47–0.92]; *P* = 0.053; *Cox test P* = 0.016), indicating that TTFields therapy may reduce the risk of death by 34% vs PC-SOC. Interestingly, time to treatment failure was improved with TTFields therapy vs PC-SOC (3.3 vs 1.6 months, respectively; *log-rank test*, *P* < 0.0001), and OS was extended for the PP population (8.1 vs 6.4 months) in the TTFields therapy vs PC-SOC arms, respectively (key additional endpoint; HR [95% CI], 0.60 [0.42–0.85]; *P* = 0.017; *Cox test P* = 0.004).

TTFields therapy vs PC-SOC, respectively, showed increases in 1-year OS rates of 43% for the ITT (33% vs 23%) and 52% for the PP (35% vs 23%) populations, suggesting improved long-term survival benefit with TTFields therapy. Patients treated with TTFields therapy who met the usage goal of ≥ 18 h/day (≥ 75% daily usage) had higher OS rates than PC-SOC-treated patients at 12 (42% vs 23%) and 18 (27% vs 18%) months. A similar suggestion of improved survival with usage over time of ≥ 18 h per day has been observed for patients with ndGBM treated with TTFields therapy (EF-14 study) [42].

The findings of the present study support the findings of a noninferiority analysis of the EF-11 overall survival data, which indicated that TTFields therapy may be at least equivalent to active chemotherapy [32]. Interestingly, the proportion of patients who had at least one cycle of treatment was higher in this real-world population compared with patients receiving TTFields monotherapy in EF-11 (95% vs 78%). This registry study was initiated following the approval of TTFields therapy for GBM, by the FDA, which may have increased patient/physician acceptance and level of usage to the treatment, contributing to the increased treatment exposure.

A weakness of this study is that the comparator group is a historical control (as is typical of a registry study), although it is used to compare relative to a prospectively collected data set. Furthermore, real-world studies inherently lack the robustness of standardized randomized controlled trials (RCTs) and may be subject to a level of selection and interpretation bias not associated with more tightly controlled RCTs. As such, baseline demographic variations between treatment groups, such as the unavailability of KPS score for approximately one-third of study group patients, could lend potential bias to the results. Nevertheless, real-world evidence and RCT data are mutually complementary, and the real-world data presented here are in line with and lend further support to the data published in the EF-11 study.

Although differences in baseline prognostic factors may impact the results of the comparison of outcomes, we note there was a trend of observed benefit with use of TTFields therapy for rGBM treatment across the evaluated subgroups in the ITT population. Patient characteristics that were associated with maximal benefit with TTFields therapy vs PC-SOC, respectively, were < 65 years of age (8.2 vs 6.5 months; *log-rank test P* = 0.05 and KPS of 90–100 (14.2 vs 8.6 months; *log-rank test P* = 0.053).

There was clinical benefit with TTFields therapy vs PC-SOC, respectively, in patients with *MGMT* promotor methylation (10.0 vs 6.5 months) and with *IDH1* gene mutation (14.4 vs 6.5 months) statuses. The benefit observed in patients with *MGMT* non-methylated promotor status and extended benefit in methylated promoter status [43] suggests broad utility of TTFields therapy in these subgroups, as monotherapy and concomitant with TMZ [44], especially in the non-methylated promoter subgroup with limited treatment options [45]. Previous data have shown benefit with TTFields therapy in patients with *IDH1* wild-type ndGBM tumors [31]. The data obtained from the present study lends further support to the previous findings in *IDH1* wild-type tumors, with probable extended benefit in *IDH1* mutation-positive patients.

As expected, TTFields therapy was well tolerated with a favorable safety profile compared with PC-SOC. The most common treatment-related AEs were manageable and resoluble local skin AEs at array site of contact rather than systemic, less-tolerable chemotherapy-related AEs. Beneath-array localized skin AEs associated with TTFields therapy may include contact dermatitis, hyperhidrosis, pruritus, skin erosion, or ulceration [41], and can typically be managed by practical approaches including optimal shaving and shifting the array position (~ 2 cm), or by the use of early pharmaceutical interventions [41].

Patients receiving PC-SOC experienced a higher rate of chemotherapy-related AEs (e.g., nervous system, gastrointestinal, hematological, metabolic, and infectious) vs those receiving TTFields therapy. The reported incidence of neurological symptoms across both treatment arms was expected, due to known-reported underlying GBM disease burden, including headaches, seizures, and cognitive changes [41, 46]. Importantly, TTFields therapy did not increase the incidence of such neurological AEs.

## Conclusions

In this EF-19 prospective, post-marketing registry study of adult patient population with rGBM, TTFields monotherapy (200 kHz) demonstrated efficacy and safety, providing support to the phase III EF-11 study findings. Patients who received TTFields monotherapy showed comparable OS in the ITT population, improved OS in the PP population, and a more tolerable/favorable safety profile vs PC-SOC in this real-world setting. No new safety signals or increases in systemic side effects were observed. Therefore, these data provide additional supportive evidence of the efficacy and safety of TTFields therapy as an appropriate therapy for rGBM patient population. Furthermore, as in other previously reported studies demonstrating the clinical effectiveness of TTFields therapy, continuous use of TTFields therapy for ≥ 18 h per day (over a 4-week average) was associated with better survival outcomes vs lower usage time.

## Supplementary Information

Additional file1 (DOCX 23 KB)

## Data Availability

The datasets generated and/or analyzed during the current study are available 3 years after date of publication.
