# Supplementary material for: Tumor Treating Fields (TTFields) therapy vs physicians’ choice standard-of-care treatment in patients with recurrent glioblastoma: a post-approval registry study (EF-19)
Source: Discov Oncol. 2022 Oct 14;13:105. doi: 10.1007/s12672-022-00555-5 (PMC9568629; doi:10.1007/s12672-022-00555-5)
Supplement: Supplementary file 1 — Additional file1 (DOCX 23 KB) [file 12672_2022_555_MOESM1_ESM.docx]

# Supplementary Information

**Inclusion criteria**

- Aged ≥ 22 years of age
- Histological diagnosis of glioblastoma (World Health Organization grade IV)
- Tumor located in the supra-tentorial region of the brain
- Received maximal, safe, surgical resection (further resection upon recurrence was permitted; however, information regarding re-irradiation was not collected)
- Received maximal radiation therapy (45–70 Gy)
- Received concomitant temozolomide (75 mg/m^2^/day for 6 weeks)
- Received maintenance temozolomide (150–200 mg/m^2^ daily for 5 days followed by 23 days without treatment, for 6 cycles or until disease progression)
- Karnofsky Performance Score ≥ 70
- Women of childbearing age must be on effective contraception
- Signed informed consent

**Exclusion criteria**

- Implanted electronic medical device in the brain:
  - Deep brain stimulator
  - Vagus nerve stimulator
  - Programmable shunt
- Skull defect without replacement
- Receiving concomitant chemotherapy
- Unable to comply with treatment with Optune
- Pregnant
- Actively participating in another therapeutic clinical trial
- Radiological suspicion of pseudoprogression or radionecrosis (a cold PET scan or negative biopsy are required in order to rule out these conditions if radiological suspicion exists)
- Any serious co-morbidity which is expected to affect survival more adversely than glioblastoma
